# The Impact of Cleft Lip/Palate and Surgical Intervention on Adolescent Life Outcomes

**DOI:** 10.5334/aogh.3679

**Published:** 2022-04-13

**Authors:** Bruce Wydick, Mustafa Zahid, Sam Manning, Jeremiah Maller, Kira Evsanaa, Susann Skjoldhorne, Matthew Bloom, Abhishek Das, Gaurav Deshpande

**Affiliations:** 1 University of San Francisco, 2130 Fulton Street, San Francisco, US; 2 Center for Effective Global Action (CEGA), and Distinguished Research Affiliate, University of Notre Dame, US; 3 Center on Food Security and the Environment, Stanford University, US; 4 OpenResearch, US; 5 Apple Computer, Inc., US; 6 Capital One, San Francisco, US; 7 WestEd, US; 8 Mendoza College of Business, University of Notre Dame, US; 9 Mahatma Gandhi Medical College, IN

## Abstract

**Background:**

Cleft lip/palate (CLP) is a congenital orofacial anomaly appearing in approximately one in 700 births worldwide. While in high-income countries CLP is normally addressed surgically during infancy, in developing countries CLP is often left unoperated, potentially impacting multiple dimensions of life quality. Previous research has frequently compared CLP outcomes to those of the general population. But because local environmental and genetic factors contribute to the risk of CLP and also may influence life outcomes, such studies may downwardly bias estimates of both CLP status and correction.

**Objectives:**

This research represents the first study to use causal econometric methods to estimate the effects of both CLP status and CLP correction on the physical, social, and mental well-being of children.

**Methods:**

Data were collected first-hand from 1,118 Indian children, where we obtained first-hand data on height, weight, grip strength, cognitive ability, reading, and math ability. A professional speech therapist reviewed digital recordings of speech taken at the interview to obtain four measures of speech quality. Using this data, the household fixed-effects model we employ jointly estimates effects of CLP status and CLP surgical intervention.

**Findings:**

Our results indicate that adolescents with median-level CLP severity show statistically significant losses in indices of speech quality (-1.59σ), physical well-being (0.32σ), academic and cognitive ability (-0.37σ), and social integration (-0.32σ). We find strong evidence that CLP surgery significantly restores speech if performed before five years of age. The first surgeries performed on less-severe CLP cases significantly restore social integration, psychological well-being, academic/cognitive ability, and a general index of human flourishing.

**Conclusions:**

Children born with CLP in India face statistically significant losses in speech, physical health, mental health, and social inclusion. CLP surgical intervention significantly restores speech quality if carried out at an early age. Surgeries with the most significant impact on life outcomes are the first surgeries performed on less-severe CLP cases.

## 1. Introduction

One out of about 700 children in the world are born with cleft lip, cleft palate, or both (CLP). CLP is a craniofacial abnormality with a prevalence rate varying across geographical areas, ethnic and socioeconomic groups, and genders [1,2,3]. It is believed to result in significant disadvantages in later life, which may include effects on speech [4], physical development [5], psychological health [6,7,26,30], cognition and learning [8,9,28,29], bullying [6] and social exclusion [8].

In high-income countries, those born with CLP generally enjoy access to corrective surgeries and undergo reparative surgery after the first few months of birth with follow-up surgeries in later years [31]. However, in low- and middle-income countries (LMICs) surgical care for CLP is often limited, especially in rural areas [10]. These factors often lead to treatment delays causing large numbers of untreated patients. In India, for example, backlogs of CLP patients have been reported to be as high as one million [11,12]. Global non-profit organizations have sought to fill this gap, with Smile Train and Operation Smile the most well-known of these specializing in CLP surgery.^1^

Research on the effects of CLP and its corrective surgeries typically compares well-being measures of individuals with CLP to the general population. However, CLP is caused by a complex interaction of genetic, syndromic, familial, and local environmental factors, including maternal factors affecting fetal development: maternal smoking and exposure to second-hand smoke, deficiencies in vitamin A, vitamin B6, riboflavin, and zinc, exposure to organic solvents and agricultural chemicals, and maternal stress [3,25,27]. Consequently, studies that use average outcomes from the general population as a counterfactual to CLP status, even when subjects are matched by gender and age, are likely to produce (downwardly) biased estimates of both the impact of CLP itself and corrective surgeries because factors that are correlated with higher rates of CLP are likely to yield low outcomes irrespective of CLP status. As a result, quasi-experimental methods are critical to this type of analysis, but they have not been effectively used to date to estimate causal impacts of CLP and CLP interventions. This study seeks to fill this gap.

## 2. Methods

### Data Sources and Participants

Our survey contains 1,118 subjects, 552 of which were in families with a CLP child. The remaining subjects were in families without a CLP child and are used as additional controls. Of our 276 CLP adolescents age 11–19, 238 had received at least one surgery, and 38 of the CLP children were completely unoperated. To generate counterfactual outcomes for CLP status, we also surveyed the nearest-age sibling^2^ of the CLP subject, which account for another 276 of our subjects. In addition to the 552 observations (276 CLP children and 276 of their nearest-age siblings), we also surveyed 283 pairs of siblings (566 total observations) in the same age range from randomly surveyed non-cleft households within 36 randomly selected villages in the regions in which all of the CLP subjects and nearest-age siblings live.^3^ Consent and assent were obtained from subjects and their guardians (see Appendix).

We partnered in our research with Operation Smile (OS),^4^ one of the two widely known non-profit organizations performing CLP surgeries internationally. Data collection ran continuously from May 2017 to June 2019. While domestic hospitals and other international non-profits, including Smile Train, use local surgeons, OS often flies surgeons into countries where it works (such as India) to carry out surgical missions lasting between one and three weeks and carries out a more comprehensive psychosocial intervention than most other CLP surgical providers.

**Figure 1:**
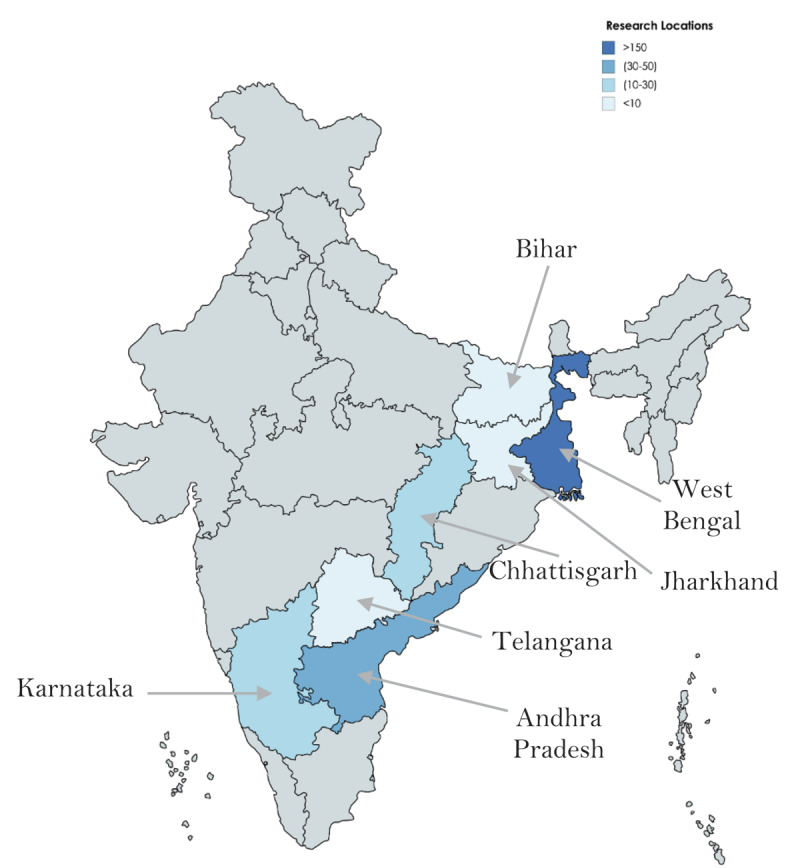
Areas of data collection, color-coded by number of subjects.

We collected data on two types of adolescents born with CLP: 1) past CLP patients of OS, all of whom had received at least one CLP surgery; and 2) future patients of OS, some of whom were unoperated, while others had received at least one previous CLP surgery. Our survey took place in the Indian states of West Bengal, Andhra Pradesh, Telangana, Karnataka, and Chhattisgarh (see ***Figure 1***). To obtain data on CLP subjects without cleft surgery, we collected data from subjects at OS screening camps and current CLP missions before they were scheduled to receive treatment. Multiple surgeries are required to fully treat CLP, and many CLP subjects that were surveyed at OS screening camps and missions had previously received surgery from OS and/or another provider. In order to estimate the differential impact of receiving surgery from OS versus other providers, we collected data about the number of past surgeries received and whether or not treatment was provided by OS. In all cases, pairs of siblings were surveyed in the same location to ensure that the location of the survey had no confounding influence on differences in survey responses among siblings.

We collected first-hand data on height, weight, and grip strength, administered digit-span memory tests to measure cognitive ability, and carried out reading and math evaluations using questions from the 2016 nationwide Annual Statistics of Education Report (ASER) survey. Enumerators captured digital speech recordings taken at the interview to measure speech anomalies where subjects read from a standard text and recited a series of common numbers. The speech in these recordings was then reviewed by a professional speech therapist for hypernasality, hyponasality, air emission/turbulence, understandability, and social acceptability.

Since our unit of programmatic intervention is the CLP surgery, we classify each CLP subject in our study according to cleft severity by the estimated number of surgeries required to restore the patient to physical “near normalcy” in terms of appearance and physical restoration.^5^ From a long record of CLP surgical interventions, we categorized each CLP subject based on the estimated number of surgeries required to restore a child born in the corresponding condition to physical near normalcy:

1. Incomplete unilateral or bilateral cleft lip, but no cleft palate: 2 surgeries
2. Incomplete unilateral or bilateral cleft palate, but no cleft lip: 3 surgeries
3. Complete unilateral or bilateral cleft lip: 4 surgeries
4. Incomplete cleft lip (bi/unilateral) and incomplete cleft palate (bi/unilateral): 5 surgeries
5. Complete unilateral cleft lip and palate: 6 surgeries
6. Complete bilateral cleft lip and palate: 7 surgeries
7. Complete bilateral cleft lip and palate with deviated premaxilla: 8 surgeries

Based on these estimates, the average individual in our Indian sample born with CLP requires 4.53 surgeries for restoration to physical near normalcy.

Using the method of Kling et al., [13] we created indices for broad categories of life outcomes: 1) speech, 2) overall physical abilities, 3) psychological health, 4) social integration, 5) cognitive and academic ability, and 6) an overall human flourishing index. The human flourishing index is a summary index of all of these indices, equally weighted, and similarly standardized.

### Quasi-Experimental Design

Because CLP surgical intervention cannot be ethically randomized, we implement a quasi-experimental methodology in our research. Using a household-level fixed effect with data on nearest-age siblings, we are able to estimate the average effect of CLP status by implicitly comparing the differences in outcomes between *unoperated* CLP children (by degree of severity) and their age-proximate sibling. This allows for control of family and environmental characteristics shared by siblings in which the non-CLP sibling generates a counterfactual for life outcomes in the absence of CLP, controlling for age, gender, and birth order. The impact of surgical interventions is given by implicitly subtracting the difference between *operated* CLP children and their non-CLP siblings from the difference between *unoperated* CLP children and their own non-CLP siblings. Use of the household fixed effect allows us to control for unobservable factors at the family level that may influence selection into surgery as well as an array of life outcomes across siblings.

We make four key assumptions as a basis for our fixed-effects model based on a current understanding of the causes of CLP. First, conditional on household environment and maternal factors, we assume that CLP status occurs randomly across siblings [14,15,16]. Second, CLP surgery is random conditional on household characteristics (which are held constant via the household fixed-effect.) Third, the expected difference in potential outcomes for CLP subjects and siblings conditional on gender, age, and birth order is constant. Last, we assume that the CLP status and surgical status of one sibling does not affect the potential outcomes of the other sibling.

A key assumption is the absence of spillovers between siblings from both CLP status and subsequent surgeries. To the extent that CLP status imposes negative spillover effects on the life outcomes of non-CLP siblings, our estimates underestimate the impacts of CLP but do not affect the estimates of the average treatment effect of the surgeries. Similarly, if there were positive spillovers to non-CLP siblings from surgical intervention on the CLP sibling, it would result in an underestimate of the effects of CLP treatment. We do not believe these spillovers significantly affect our estimates: We carry out robustness check estimations using regional-level fixed effects (shown in Appendix Table A1) that incorporate children outside the household for whom any spillovers would more obviously be negligible. These regional fixed-effect results are substantially consistent with our preferred model, which we favor because it more tightly controls for family and household background and environment.

### Statistical Model

We thus estimate the following model:


1 {y_{ij}} = \alpha + {\beta _1}{C_i} + {\beta _2}{S_i} + \omega O{S_i} + {{\boldsymbol X_{ij}}^{\prime} {\theta}} + {\mu _j} + {\varepsilon _{ij}}

where *y_ij_* is the outcome index *y* for person *i* in household *j, C_i_* is a variable representing the severity of CLP as measured by the number of surgeries needed at birth to physically restore the individual to physical near-normalcy, *S_i_* are the number of reparative CLP surgeries performed on the individual, *OS_i_* is a variable for the number of surgeries performed specifically by Operation Smile (which we include in a second summary index estimation), ***X_ij_*** is a vector of control variables that include gender, age, birth order that will be used to distinguish child *i* in household *j, μ_j_* is a household level fixed effect, and *ɛ_ij_* is the error term.^6^ When we estimate our model with *regional* fixed effects from the 36 regions (groups of proximate villages) in our data, we add household controls that include a dummy variable indicating whether a household has a CLP child, education and occupation of parents, a housing quality index, and dummy variables indicating if a household is Christian or Muslim (the default being Hindu).

The hypotheses from our research were developed in a publicly available pre-analysis plan prior to our fieldwork.^7^ The coefficients of greatest interest in these regressions are 𝛽_1_ and 𝛽_2_, representing the impact of cleft severity (in terms of required surgeries) and the impact of corresponding received surgeries, respectively. Our null hypotheses are that cleft severity has no impact on our life outcome variables, and receiving reparative surgeries has no impact on restoration of life outcomes (𝛽_1_ = 𝛽_2_ = 0), with the alternatives being that 𝛽_1_ < 0 and 𝛽_2_ > 0. Within this framework we can also test a null hypothesis that CLP surgery fully restores a given life outcome index, *i.e*., 𝛽_1_ + 𝛽_2_ = 0, rejecting the null if CLP outcomes remain significantly negative 𝛽_1_ + 𝛽_2_ < 0 even after surgery.

## 3. Results and Discussion

The descriptive statistics in ***Table 1*** reveal noticeable differences in outcomes across outcome categories, with unoperated CLP adolescents faring worse in terms of social integration, psychological well-being, academic and cognitive abilities, and in our human flourishing index, which places equal weights on each of our indexed categories. Operated CLP adolescents are superior across these outcomes, but still rank below their age-proximate siblings.

**Table 1:** Descriptive Statistics: India CLP Data.


	UNOPERATED CLP ADOLESCENTS		OPERATED CLP ADOLESCENTS		NON-CLP ADOLESCENTS	
		
	CLP ADOLESCENT	SIBLING	CLP ADOLESCENT	SIBLING	ALL NON-CLP ADOLESCENTS	NON-CLP HH ADOLESCENTS

Male	0.579	0.579	0.492	0.555	0.522	0.504

	(0.081)	(0.081)	(0.032)	(0.032)	(0.016)	(0.022)

Age	14.421	13.132	14.445	14.441	14.027	13.533

	(0.398)	(0.687)	(0.170)	(0.305)	(0.119)	(0.142)

Birth Order	2.447	2.447	1.920	2.042	2.035	1.975

	(0.225)	(0.232)	(0.071)	(0.061)	(0.033)	(0.042)

Physical Well-being	0.004	–0.019	–0.036	0.166	0.020	–0.083

	(0.122)	(0.156)	(0.050)	(0.061)	(0.026)	(0.032)

Social Integration	–0.074	0.015	–0.064	0.007	0.017	0.043

	(0.083)	(0.069)	(0.029)	(0.029)	(0.014)	(0.018)

Psychological Well-being	–0.219	–0.002	–0.089	0.000	0.012	0.068

	(0.084)	(0.067)	(0.036)	(0.031)	(0.015)	(0.018)

Academic and Cognitive Abilities	–0.599	–0.191	–0.127	0.044	0.024	0.127

	(0.126)	(0.139)	(0.055)	(0.054)	(0.024)	(0.029)

Human Flourishing Index	–0.135	–0.006	–0.032	0.062	0.011	0.000

	(0.050)	(0.056)	(0.023)	(0.023)	(0.010)	(0.013)

N	38	38	238	238	982	522


### Speech Outcomes

Our estimations in ***Table 2*** (panel A) show that for every unit of cleft severity (given in terms of surgeries needed to restore physical near-normalcy) speech quality declines by 0.28σ with respect to hypernasality, 0.25σ for hyponasality, 0.28σ for turbulence during vocal air emission, and 0.31σ for understandability, and there is a 0.30σ reduction in social acceptability of speech.^8^ Overall, our aggregated speech index measure falls by 0.35σ. Because the average number of surgeries needed at birth is 4.53, we estimate that the CLP disability causes a decline in speech quality of 1.59σ below the counterfactual age-proximate sibling outcome.

**Table 2:** The Impact of cleft severity and cleft surgeries on speech outcomes.


*PANEL A:*	OVERALL SPEECH INDEX^1^	OVERALL SPEECH INDEX^2^	HYPER-NASALITY	HYPO-NASALITY	AIR EMISSION	UNDER-STANDABILITY	ACCEPTABILITY

Cleft Severity	–0.351***	–0.345***	–0.280***	–0.247***	–0.284***	–0.305***	–0.296***

	(0.0349)	(0.0353)	(0.0346)	(0.0624)	(0.0452)	(0.0322)	(0.0346)

Cleft Surgeries	–0.0374	–0.00981	–0.161**	0.235**	–0.00767	–0.0392	–0.0101

	(0.0674)	(0.0715)	(0.0707)	(0.118)	(0.0892)	(0.0596)	(0.0715)

Operation Smile surgeries		–0.137					

		(0.112)					

*N*	954	954	926	925	921	926	925

**IMPACT OF EARLY SURGERY MATTER ON SPEECH OUTCOMES:**							

***PANEL B:***	**OVERALL SPEECH INDEX**	**OVERALL SPEECH INDEX**	**HYPER-NASALITY**	**HYPO-NASALITY**	**AIR EMISSION**	**UNDER-STANDABILITY**	**ACCEPTABILITY**

Cleft Severity	–0.365***	–0.359***	–0.281***	–0.256***	–0.313***	–0.329***	–0.316***

	(0.0361)	(0.0363)	(0.0359)	(0.0676)	(0.0475)	(0.0328)	(0.0364)

Cleft Surgeries	–0.0913	–0.0629	–0.165**	0.206*	–0.0926	–0.114*	–0.0730

	(0.0733)	(0.0757)	(0.0762)	(0.119)	(0.0962)	(0.0678)	(0.0754)

Operation Smile Surgeries		–0.156					

		(0.113)					

First Surgery ≤ Five Years Old	0.310*	0.327*	0.0238	0.173	0.525**	0.460***	0.385**

	(0.166)	(0.168)	(0.176)	(0.295)	(0.218)	(0.168)	(0.171)

*N*	954	954	926	925	921	926	925


OLS with fixed effects at the household level. Standard errors clustered at the household level and are in parentheses. Regressions control for individual variables including gender, birth order, and age. Dependent variables are all standardized Kling et al. [13] indices. * *p* < 0.10, ** *p* < 0.05, *** *p* < 0.01. ^1^ Test of (full restoration of speech index) rejected (*p* < 0.01). ^2^ Test of (full restoration of human flourishing index with Operation Smile surgeries) rejected (*p* < 0.01).

Consistent with previous findings such as Hardin-Jones and Jones [17], D’Antonio and Scherer [4], and Mitacek [18], in ***Table 2*** (panel A), we find no overall evidence of positive impact from cleft surgeries without controlling for the age at which they occurred. CLP surgeries in our sample result in reduced hyponasality but increased hypernasality and small and insignificant effects on other speech outcomes, and we reject the hypothesis that CLP surgery fully restores speech, i.e. 𝛽_1_ + 𝛽_2_ = 0. This may be in part because few of the CLP adolescents had access to follow-up speech therapy after surgery, although we have no precise data on speech therapy for individual subjects.

**Figure 2:**
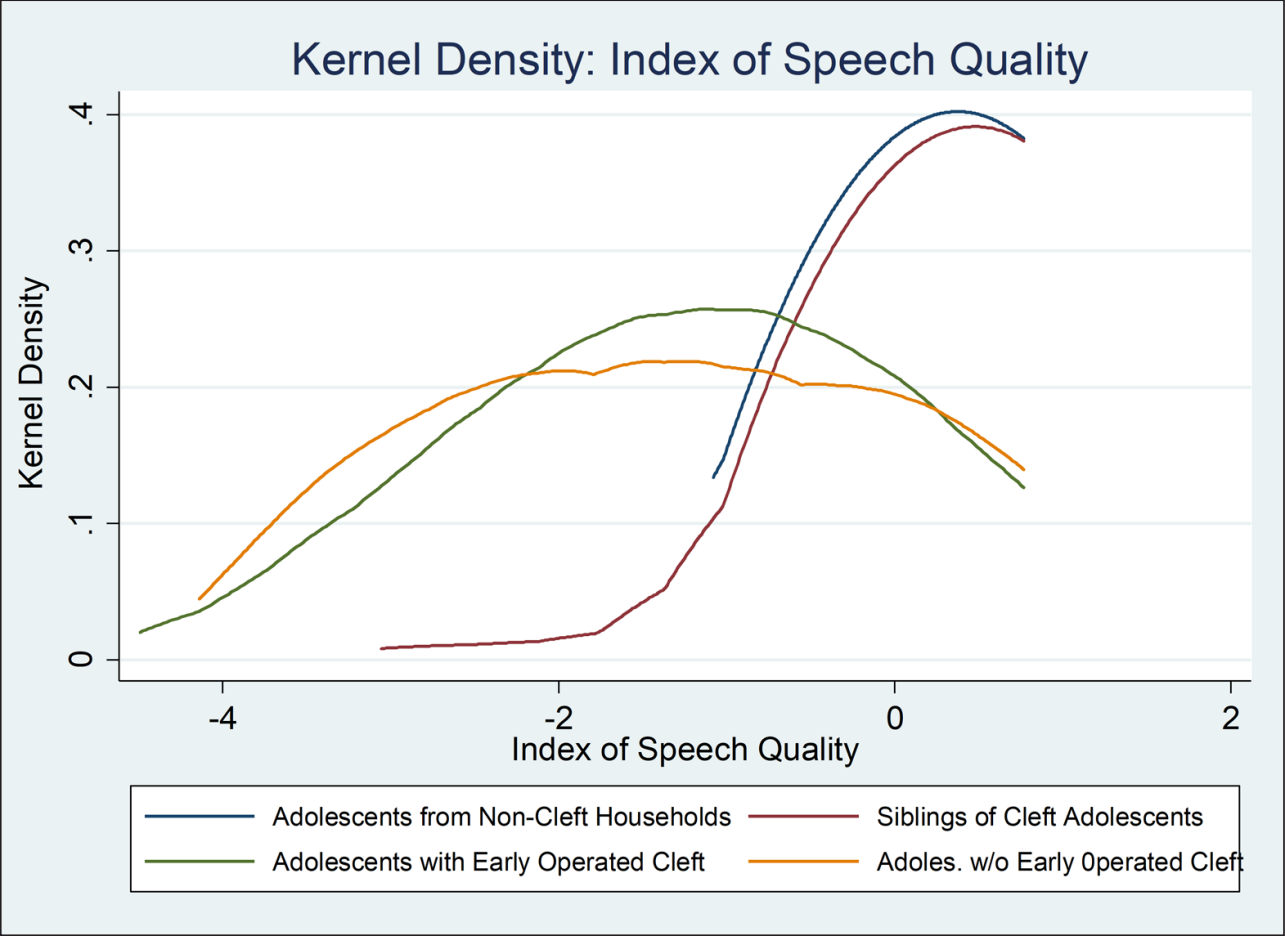
Kernel Density of Speech Quality by Cleft and Surgery Status.

However, in ***Table 2*** (panel B) our estimates show a very large and statistically significant positive impact of early surgery (£ 5 years) that is close to the negative effect of one degree of cleft severity in the overall speech quality index, but has a particularly strong effect on reducing air emission/turbulence during speech, general understandability, and social acceptability of speech.^9^ We show a kernel density function of the differences between early operated and non-early operated CLP subjects in ***Figure 2***. Our results on speech clearly support previous research suggesting that early-age surgeries have substantially greater impacts on speech quality. Estimates using regional fixed effects for speech and other outcomes show similar results and are given in the Appendix in Table A1.

### Physical Outcomes

Infants with CLP can have feeding difficulties, possibly leading to malnutrition at an early stage that can affect the healthy growth children born with CLP as a result of lower caloric intake [19,20,24], where lower BMI is a common outcome of CLP. We obtained measures of height, weight, and grip strength from subjects in our study as well as perceived physical well-being, which included questions to subjects about difficulties carrying out physical tasks such as daily chores, eating, and drinking. ***Table 3*** provides estimates which show that CLP adolescents register 0.072σ lower in overall physical well-being than the outcomes of their nearest-age sibling, and 0.135σ lower in perceived physical well-being. While there is no difference in grip strength, CLP adolescents are 0.082σ lower based on a BMI index calculated through a weight-to-height ratio. Our estimations find CLP surgery to have insignificant effects on overall physical measures, showing a 0.02σ improvement in physical well-being and a 0.05σ improvement in perceived physical well-being that are both statistically insignificant.^10^

**Table 3:** The Impact of cleft severity and cleft surgeries on physical and health outcomes (Household Fixed Effects).


	OVERALL PHYSICAL WELLBEING^1^	OVERALL PHYSICAL WELLBEING^2^	OVERALL PERCEIVED PHYSICAL WELLBEING	WEIGHT FOR HEIGHT	GRIP STRENGTH

Cleft Severity	–0.0722***	–0.0742***	–0.135***	–0.0820***	–0.0167

	(0.0213)	(0.0212)	(0.0345)	(0.0316)	(0.0203)

Cleft Surgeries	0.0369	0.0215	0.0968	0.0449	–0.0218

	(0.0442)	(0.0502)	(0.0674)	(0.0465)	(0.0371)

Operation Smile Surgeries		0.0707			

		(0.0794)			

*N*	1118	1118	1118	1118	1118


OLS with fixed effects at the household level. Standard errors clustered at the household level and are in parentheses. Regressions control for individual variables including gender, birth order, and age. Dependent variables are all standardized Kling et al. [13] indices. * *p* < 0.10, ** *p* < 0.05, *** *p* < 0.01. ^1^ Test of (full restoration of physical outcomes index at early surgery) not rejected (*p* = 0.21). ^2^ Test of (full restoration of physical outcomes index) not rejected (*p* = 0.78).

### Social Integration

**Figure 3:**
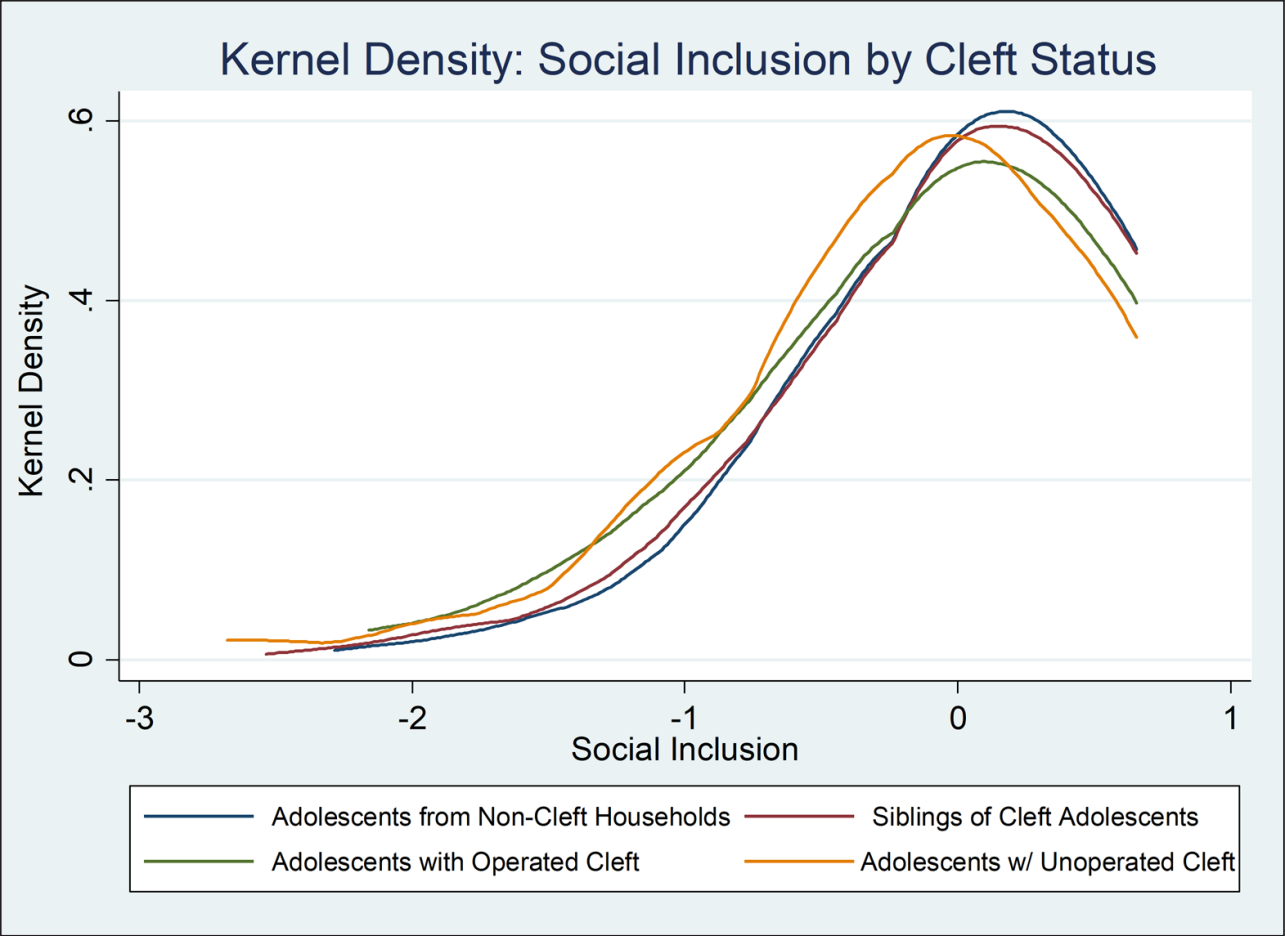
Kernel Density of Social Inclusion by Cleft Status.

The two components of social integration in our research are *social inclusion*, the degree to which a person is able to form relationships with community, and *social behavior*, the degree to which one’s behavior adheres to appropriate social norms. In ***Table 4*** we find significant negative effects from CLP status on our index of social integration (–0.072σ), implying that the level of social integration for the average CLP subject in our study is 0.32σ below that of the age-proximate sibling counterfactual. Essentially all of this effect is driven from a lower level (–0.089σ), of social inclusion, which is in turn driven largely by a CLP adolescent’s lack of freedom from bullying and teasing (–0.089σ). ***Figure 3*** gives density functions of social inclusion by cleft status, showing the lower level of social inclusion experienced by CLP children. We also find that while evidence from high-income countries suggests that parents allocate more time to children with disabilities [21], we actually find negative (but statistically insignificant) estimates from CLP status on a parental support index, indicating that CLP children do not receive the extra time allocation from their parents required to meet the special needs of CLP children.

**Table 4:** The Impact of cleft severity and cleft surgeries on social integration (Household Fixed Effects).


	SOCIAL INTEGRATION^1^	SOCIAL INTEGRATION^2^	SOCIAL INCLUSION	PROSOCIAL BEHAVIOR	FREEDOM FROM BULLYING	PARENTAL SUPPORT

Cleft Severity	–0.0716**	–0.0648*	–0.0887***	–0.0202	–0.0891***	–0.0184

	(0.0347)	(0.0345)	(0.0340)	(0.0368)	(0.0309)	(0.0327)

Cleft Surgeries	0.0691	0.120*	0.0932	0.0102	0.0605	–0.0227

	(0.0667)	(0.0687)	(0.0666)	(0.0730)	(0.0608)	(0.0620)

Operation Smile Surgeries		–0.233**				

		(0.119)				

*N*	1118	1118	1118	1118	1118	1118


OLS with fixed effects at the household level. Standard errors clustered at the household level and are in parentheses. Regressions control for individual variables including gender, birth order, and age. Dependent variables are all standardized Kling et al. [13] indices. * *p* < 0.10, ** *p* < 0.05, *** *p* < 0.01. ^1^ Test of (full restoration of social integration) not rejected (*p* = 0.95). ^2^ Test of (full restoration of social integration index) not rejected (*p* = 0.07).

The estimates for impacts on social integration from CLP surgery are positive, though statistically insignificant. However, we cannot reject the null hypotheses (𝛽_1_ + 𝛽_2_ = 0) that CLP surgery fully restores social integration (*p* = 0.95) through its effect on reduced bullying and in turn increasing social inclusion. In Appendix Table A2, we examine mediators that affect social inclusion, where somewhat surprisingly we find that it is speech quality that affects social inclusion more than the outward appearance of a visible cleft lip. The table shows that a one-standard-deviation increase in speech quality increases social inclusion by 0.25σ (*p* < 0.01), whereas the other factors, including an unoperated (and hence visible) cleft lip are insignificantly related.

### Psychological Well-Being

We created indices from our psychological questionnaire for depression, anxiety, hope, and self-esteem, and we find CLP adolescents to have lower outcomes in each of these areas although none retain statistical significance.^11^ We find CLP status to cause a 0.047σ (*p* = 0.07) reduction in overall psychological well-being in our specification in column 2 of ***Table 5***. This implies that the average CLP subject falls 0.22σ below the counterfactual age-proximate sibling in psychological health. We do not find evidence that CLP surgery has a restorative effect on psychological well-being generally, but our estimates show that OS surgeries appear to produce better results in this outcome than other CLP surgeries, and we cannot reject the null hypothesis that psychological well-being is fully restored with OS surgeries, a difference we will discuss below.

**Table 5:** The Impact of cleft severity and cleft surgeries on psychological well-being (Household Fixed Effects)


	OVERALL PSYCHOLOGICAL WELL-BEING^1^	OVERALL PSYCHOLOGICAL WELL-BEING^2^	DEPRESSION	ANXIETY	HOPE	SELF-ESTEEM

Cleft Severity	–0.0373	–0.0468*	–0.0339	–0.0259	–0.00445	–0.0346

	(0.0280)	(0.0284)	(0.0328)	(0.0327)	(0.0301)	(0.0257)

Cleft Surgery	–0.0285	–0.0129	–0.00126	–0.00112	–0.0942	0.0407

	(0.0537)	(0.0688)	(0.0719)	(0.0683)	(0.0627)	(0.0491)

Operation Smile Surgeries		0.137				

		(0.123)				

*N*	1118	1118	1118	1118	1118	1118


OLS with fixed effects at the household level. Standard errors clustered at the household level and are in parentheses. Regressions control for individual variables including gender, birth order, and age. Dependent variables are all standardized Kling et al. [13] indices. * *p* < 0.10, ** *p* < 0.05, *** *p* < 0.01. ^1^ Test of (full restoration of psychological well-being) rejected (*p* = 0.04). ^2^ Test of (full restoration of psychological well-being) not rejected (*p* = 0.42).

### Academic and Cognitive Ability

**Figure 4:**
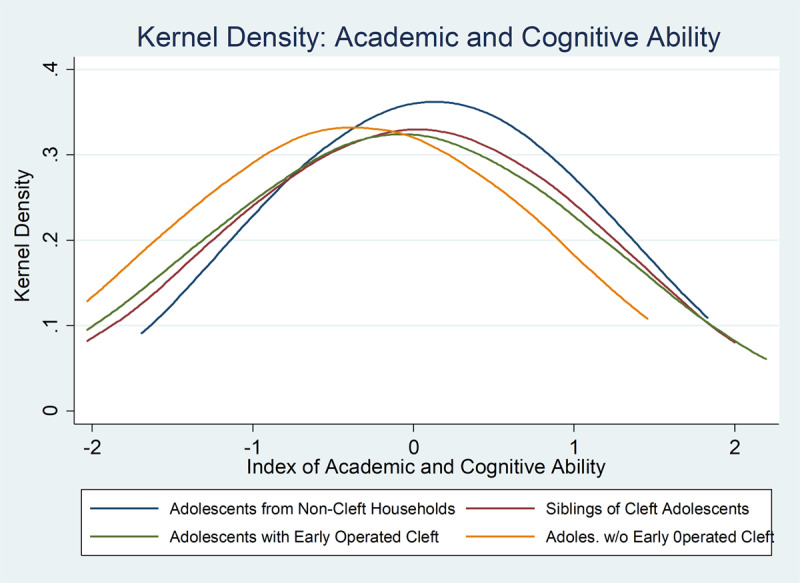
Kernel Density of Academic and Cognitive Ability.

One of the most consistent and precisely measured findings of our research is the lower academic and cognitive ability of CLP adolescents as measured by a performance on a sequence of increasingly difficult math problems, a reading exercise, and a digit-span memory test (in which subjects need to repeat an increasingly longer sequence of digits read to them). ***Table 6*** shows CLP adolescents scored 0.075σ lower than the sibling counterfactual in math ability, 0.066σ lower reading ability, and 0.063σ lower on the digit-span memory test. Overall, the index on academic and cognitive ability was lower for CLP adolescents by 0.082σ (all *p* < 0.01 and surviving over-testing corrections). This difference is illustrated in the density functions presented across cleft status in ***Figure 4***. Successive CLP surgeries do not fully reduce this gap, but we cannot reject the hypothesis (𝛽_1_ + 𝛽_2_ = 0) of full surgical restoration of academic and cognitive ability (*p* = 0.33).

**Table 6:** The Impact of cleft severity and cleft surgeries on Academic and Cognitive Ability (Household Fixed Effects).


	ACADEMIC AND COGNITIVE ABILITIES^1^	ACADEMIC AND COGNITIVE ABILITIES^2^	ACADEMIC ABILITIES	DIGIT SPAN MEMORY TEST	MATH ABILITIES	READING ABILITIES

Cleft Severity	–0.0820***	–0.0888***	–0.0821***	–0.0630***	–0.0749***	–0.0661**

	(0.0273)	(0.0279)	(0.0290)	(0.0238)	(0.0286)	(0.0284)

Cleft Surgeries	0.0500	–0.000992	0.0648	0.0245	0.0579	0.0533

	(0.0545)	(0.0533)	(0.0614)	(0.0445)	(0.0580)	(0.0626)

Operation Smile Surgeries		0.235**				

		(0.109)				

N	1118		1118	1118	1118	1118


OLS with fixed effects at the household level. Standard errors clustered at the household level and are in parentheses. Regressions control for individual variables including gender, birth order, and age. Dependent variables are all standardized Kling et al. [13] indices. * *p* < 0.10, ** *p* < 0.05, *** *p* < 0.01. ^1^ Test of (full restoration of academic and cognitive abilities) not rejected (*p* = 0.33). ^2^ Test of (full restoration of academic and cognitive ability) not rejected (*p* = 0.42).

As with psychological outcomes (and physical outcomes in our regional fixed-effect estimations), effects of surgical interventions appear to be significantly larger for patients of OS surgeries. Aside from the difference that OS surgeons are typically flown in from overseas (where other providers tend to use local surgeons), there may be other reasons for this difference. First, the organization invests strongly in psychosocial intervention, assigning a psychosocial care worker to each child, and helping the child to meet other CLP children in a group before surgery so that children are able to meet and relate to others like themselves. Moreover, OS ensures that in any case in which the child presents with a cleft lip, OS always carries out this operation first. The purpose of this is that the child is able to re-integrate more quickly among peers and at school with less fear of bullying or teasing. Both of these factors may account for enhanced psychological and academic/cognitive outcomes. We find evidence for this in our regressions presented in Appendix Table A3, where we find the number of OS cleft lip surgeries to exhibit very strong and significant effects on both psychological well-being as well as in the academic/cognitive area, likely resulting from better integration with peers and schooling at an earlier age.

### Human Flourishing Index

***Table 7*** shows estimates for a human flourishing index, a summary outcome index in which we weight each of our five indexed outcomes (speech, physical, social, psychological, and academic/cognitive) equally. Here we find CLP adolescents scoring 0.083σ and 0.063σ below sibling and regional peer counterfactuals, respectively (both *p* < 0.01). Multiplying the first result by the average surgeries required, this amounts to a human flourishing loss of 0.37σ. While we reject the hypothesis that the necessary sequence of surgeries required for a given CLP case can fully restore this aggregated measure of human flourishing (*p* < 0.01), we cannot reject the hypothesis that the Operation Smile surgeries are able to fully restore this broad index of human flourishing (*p* = 0.97), again likely due to an emphasis in the psychosocial aspects of treatment.

**Table 7:** The Impact of cleft severity and cleft surgeries on Human Flourishing Index (Equality Weighted index of Outcome Summaries, Household Fixed Effects).


	HUMAN FLOURISHING INDEX^1^	HUMAN FLOURISHING INDEX^2^	HUMAN FLOURISHING INDEX	HUMAN FLOURISHING INDEX

Cleft Severity	–0.0834***	–0.0861***	–0.0629***	–0.0650***

	(0.0253)	(0.0258)	(0.0175)	(0.0176)

Cleft Surgeries	0.0109	–0.00919	–0.0223	–0.0400

	(0.0449)	(0.0473)	(0.0317)	(0.0371)

Operation Smile Surgeries		0.0924		0.0850

		(0.103)		(0.0943)

Household FE	X	X		

Regional FE			X	X

N	1118	1118	1118	1118


OLS with fixed effects at the household level. Standard errors clustered at the household level and are in parentheses. Regressions control for individual variables including gender, birth order, and age. Dependent variables are all standardized Kling et al. [13] indices. * *p* < 0.10, ** *p* < 0.05, *** *p* < 0.01. ^1^ Test of (full restoration of human flourishing index) rejected (*p* = 0.01). ^2^ Test of (full restoration of human flourishing index) not rejected (*p* = 0.97).

### Heterogeneous Effects of Subsequent Surgeries

**Figure 5:**
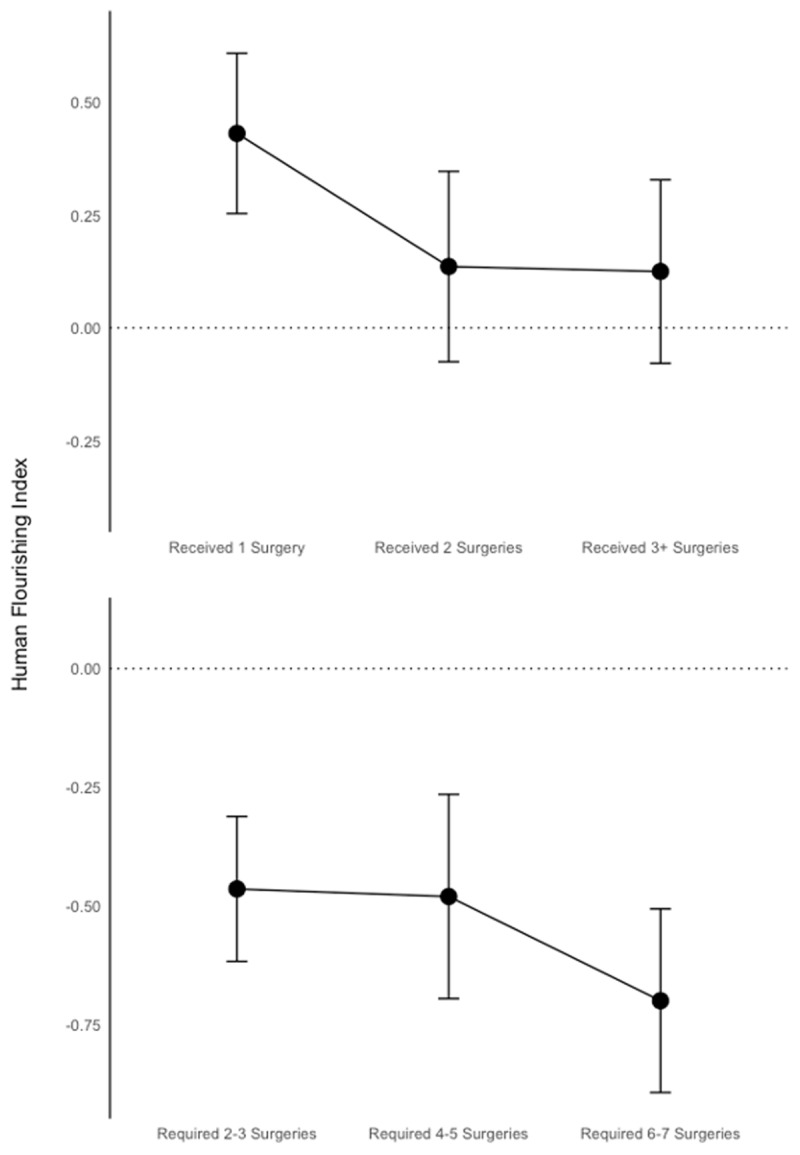
Impacts of Severity and Restoration. (Numbers of Surgeries Needed and Performed).

In ***Table 8*** we examine the effects of sequential surgeries on our different indices of adolescent life outcomes. Here, for statistical power reasons, we group surgeries into bins of 2–3, 4–5, and 6–7 required surgeries, and whether a child received 1, 2, or 3 or more surgeries. Not surprisingly we find that virtually across all indices, outcomes are increasingly worse based on the severity of the CLP condition as measured by the greater number of surgeries required to restore a child to near-normalcy. On the intervention side, there appear to be sharply higher returns to the first surgery received, where the impacts on life outcomes in our sample of high numbers of surgeries are actually negative in some cases, but strongly diminishing in others. A clear exception is in cognitive and academic outcomes, in which it is likely that a greater number of surgeries facilitates better schooling participation and (borderline significant) estimates rise slightly above 0.40σ for follow-up surgeries. ***Figure 5*** shows a graphic of the impact of surgeries, both required and needed, on the human flourishing index.

**Table 8:** Effects of Additional Surgeries: Required and Performed.


	SPEECH INDEX	PHYSICAL WELLBEING	SOCIAL INTEGRATION	PSYCHOLOGICAL WELLBEING	ACADEMIC/COGNITIVE	HUMAN FLOURISHING

Required 2–3 Surgeries	–1.373***	–0.231*	–0.244	–0.382**	–0.392**	–0.465***

	(0.259)	(0.119)	(0.226)	(0.192)	(0.183)	(0.161)

Required 4–5 Surgeries	–1.579***	–0.177	–0.462	–0.241	–0.543**	–0.481**

	(0.307)	(0.157)	(0.322)	(0.233)	(0.227)	(0.227)

Required 6–7 Surgeries	–2.156***	–0.403**	–0.439	–0.394*	–0.796***	–0.701***

	(0.314)	(0.168)	(0.284)	(0.214)	(0.244)	(0.204)

Received 1 Surgery	0.213	0.105	0.236	0.281	0.296	0.431**

	(0.280)	(0.143)	(0.258)	(0.219)	(0.200)	(0.187)

Received 2 Surgeries	0.0283	–0.0997	0.194	0.113	0.418*	0.136

	(0.332)	(0.177)	(0.299)	(0.241)	(0.248)	(0.222)

Received 3+ Surgeries	–0.268	0.0717	0.176	0.00750	0.412	0.125

	(0.329)	(0.192)	(0.285)	(0.221)	(0.251)	(0.214)

*N*	954	1118	1118	1118	1118	1118


OLS with fixed effects at the household level. Standard errors clustered at the household level and are in parentheses. Regressions control for individual variables including gender, birth order, and age. Dependent variables are all standardized Kling et al. [13] indices. * *p* < 0.10, ** *p* < 0.05, *** *p* < 0.01.

**Figure 6:**
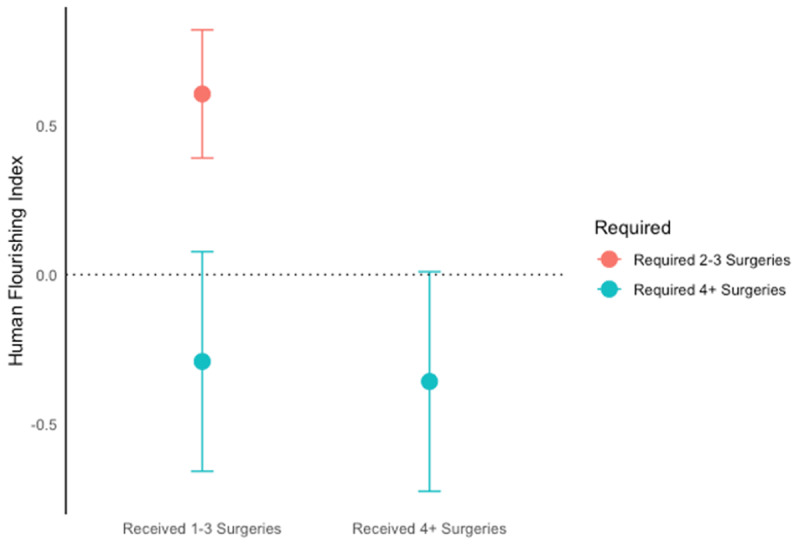
Measured Impacts of Surgeries Conditional Upon Number of Surgeries Required.

***Table 9*** shows the impact of sequential surgeries conditional on CLP severity as measured by required surgeries. These results indicate that the greatest impact is in the first surgeries performed on children requiring only 2–3 CLP surgeries. The impact of the first 1–3 surgeries on CLP patients requiring only 2–3 surgeries is given in the bottom half of the table and shows positive impacts across all indices. With the exception of physical outcomes, all of these effects are statistically significant, and the impact on the human flourishing index is 0.61σ. However, for CLP children requiring 4+ surgeries, impacts of 4+ surgeries—and even 1–3 surgeries—are statistically insignificant and in some cases have negative point estimates. A concise picture of the results on the human flourishing index is given in ***Figure 6***. What these results indicate is that CLP intervention appears to have a sharply concave shape (at best) in the life impacts of subsequent surgeries, and that the most effective surgery is the first surgery performed on a CLP child requiring only 2–3 surgeries, where subsequent surgeries—even on children who require them—show far lower impacts on adolescent life outcomes.

**Table 9:** Conditional Impacts by Varying Levels of Cleft Severity.


	SPEECH INDEX	PHYSICAL WELLBEING	SOCIAL INTEGRATION	PSYCHOLOGICAL WELLBEING	ACADEMIC/COGNITIVE	HUMAN FLOURISHING

Required 2–3 Surgeries	–1.648***	–0.196	–0.472*	–0.555**	–0.686***	–0.631***

	(0.306)	(0.129)	(0.261)	(0.241)	(0.209)	(0.184)

Required 4–7 Surgeries	–1.745***	–0.148	0.338	–0.0643	–0.270	–0.146

	(0.457)	(0.175)	(0.561)	(0.249)	(0.389)	(0.369)

Received 1–3 Surgeries	–0.950*	–0.00612	0.364**	0.0476	–0.535*	0.0936

	(0.511)	(0.315)	(0.158)	(0.317)	(0.318)	(0.294)

Received 4+ Surgeries	–2.772***	2.792***	0.618***	–1.553***	0.546***	0.982***

	(0.0827)	(0.0593)	(0.0933)	(0.0794)	(0.0712)	(0.0723)

Required 2–3 Surgeries × Received 1–3 Surgeries	1.508**	0.0308	0.187	0.439	1.264***	0.512

	(0.615)	(0.355)	(0.342)	(0.435)	(0.399)	(0.372)

Required 4+ Surgeries × Received 1–3 Surgeries	0.777	–0.174	–1.058*	–0.187	0.494	–0.385

	(0.697)	(0.371)	(0.598)	(0.421)	(0.512)	(0.485)

Required 4+ Surgeries × Received 4+ Surgeries	2.064***	–2.917***	–1.027*	1.208***	–0.755*	–1.340***

	(0.500)	(0.221)	(0.621)	(0.310)	(0.422)	(0.397)

Conditional Impacts of Surgeries on Varying Levels of Cleft Severity:						

Received 1–3 Surgeries + (Required 2–3 Surgeries × Received 1–3 Surgeries)	0.558*	0.024	0.551*	0.486*	0.729***	0.605***

	(0.343)	(0.163)	(0.303)	(0.297)	(0.242)	(0.226)

Received 1–3 Surgeries + (Required 4+ Surgeries × Received 1–3 Surgeries)	–0.172	–0.180	–0.694	–0.139	–0.041	–0.291

	(0.473)	(0.196)	(0.557)	(0.274)	(0.402)	(0.387)

Received 4+ Surgeries + (Required 4+ Surgeries × Received 4+ Surgeries)	–0.707	–0.125	–0.409	–0.345	–0.209	–0.358

	(0.048)	(0.210)	(0.603)	(0.290)	(0.415)	(0.387)

*N*	954	1118	1118	1118	1118	1118


OLS with fixed effects at the household level. Standard errors clustered at the household level and are in parentheses. Regressions control for individual variables including gender, birth order, and age. Dependent variables are all standardized Kling et al. [13] indices. * *p* < 0.10, ** *p* < 0.05, *** *p* < 0.01.

## 4. Conclusion

Causal econometrics was the subject of the 2021 Nobel Prize for Economic Sciences, but to date these methods have not been applied to understanding the effects of CLP on children or the effects of CLP interventions. Our research presents the first estimates using a causal econometric framework of the effect of CLP over a wide array of Indian adolescent life outcomes, as well as the restorative impacts of CLP surgeries. We find the adverse impacts of CLP on life outcomes to be wide-ranging, statistically significant, and large, resulting in far poorer speech, diminished physical outcomes, social exclusion, higher levels of depression, and lower cognitive ability. Our estimates indicate that CLP surgery is able to significantly restore speech quality, but only when surgery is carried out at an early age. The results strongly support previous research that has advocated for early-age CLP surgical intervention. While we do not find statistically significant effects of standard CLP surgery on many outcomes, we do find modest evidence that CLP surgery is able to restore social integration and inclusion and that early interventions carried out with a strong emphasis on the psychosocial development of CLP children appear likely to move outcomes of CLP children toward those of their age-proximate siblings. We find that the first surgeries are immensely effective at restoring human flourishing across a wide range of outcomes, especially when carried out on children who present with less-severe CLP. Thus, in a context of scarce resources where, for example only four surgeries may be funded, we find a significantly greater impact on aggregate life outcomes from performing two surgeries on each of two children who require 2–3 surgeries than performing four surgeries on one child with more severe CLP requiring more surgeries.

Previous research has demonstrated that unoperated CLP can create barriers to entering the labor market, to establishing healthy relationships, and in marriage and family formation [22,23]. As such, we view the results of this study as particularly important for the potential impacts of CLP surgery on social integration and inclusion, which may have longstanding spillover effects in later life. Our original theory of change suggested that CLP intervention would promote social inclusion through improvements in children’s appearance prior to adolescence. However, somewhat surprisingly, we find CLP speech quality to mediate social inclusion more than visual appearance. This adds further emphasis on the importance of 1) early-age surgery as a means of maximizing impacts on speech outcomes, 2) the efficacy of providing the first surgeries to children rather than concentrating higher numbers of surgeries on fewer children, and 3) the importance of providing psychosocial services to children that enable them to confidently integrate to the greatest extent possible with peers and at school.

## Additional File

The additional file for this article can be found as follows:

10.5334/aogh.3679.s1 Appendix. Additional regression tables and consent forms.

## Footnotes

Operation Smile has carried out over 220,000 surgeries since its founding in 1982, while its offshoot, Smile Train, claims 1.5 million surgeries since its founding in 1999 (*www.smiletrain.org/stories/model-empowerment*). One important difference between Operation Smile and other providers of CLP surgical intervention is that the organization has historically operated through flying surgeons from higher-income countries into surgical missions rather than strictly using the work of local surgeons.

In the event that a cleft patient either did not have a sibling or the sibling was unreachable, a patient’s nearest-age cousin was surveyed in the CLP sibling’s place if that cousin lived or was raised in the same household as the patient, the same for both control and treatment groups of CLP patient-sibling pairs. As a check on our observational data covering nearest-age siblings, we also surveyed parents for information all siblings in a household, estimations that are available on request.

This second group of subjects increases the precision of our estimates and allows for regional fixed-effect estimations that provide both a check on our household fixed-effect estimations in the possible presence of sibling externalities from CLP status.

None of the authors are affiliated with Operation Smile, although one coauthor has treated CLP patients at Operation Smile surgical missions as a volunteer surgeon.

Defined as normal orofacial functioning, full physical recovery of speech capability, and no visible cleft apart from minor surgical scarring.

Our specification imposes a linear structure on both the impact of increasing severe CLP (in terms of surgeries needed to restore near normalcy) as well as the impact of surgeries themselves. Our motivation for this is two-fold. First is that the unit of intervention is the CLP surgery for which we would like to estimate an average effect across all surgeries, and second for the purposes of increased power. We show results from a more flexible estimation in ***Table 8*** that includes dummy variables for sets of required and performed surgeries.

Our pre-analysis plan is registered with 3ie at the Registry for International Development Impact Evaluations and can be found at the URL *ridie.3ieimpact.org/index.php?r=search/detailView&id=638*.

These estimates outcomes survive the Holm-Bonferroni step-down procedure to control for over-testing that we use for our non-indexed, individual outcomes per our pre-analysis plan. We highlight those individual outcomes with significant p-values that do not survive controls for over-testing in the text.

Only about 12% of our sample was able to access CLP surgery at less than 1 year of age, and only 28% by age 2, so while point estimates are slightly higher for very early age intervention, to maximize statistical power we chose age 5 as our cutoff, which represents roughly the median age of first surgery in our sample.

We do find significant impacts on overall physical well-being in our regional fixed-effect estimations in the appendix where our measure of CLP severity by each required surgery reduced physical well-being by 0.087σ, which is restored almost exactly in these estimates by the impact each subsequent surgery 0.094σ. But because our preferred estimates find insignificant effects, we take the regional fixed-effects estimates as merely suggestive of significant effects on physical outcomes.

Estimates are given in terms for depression and anxiety such that a negative coefficient implies a worse outcome.
