# Supplementary material for: The Impact of Cleft Lip/Palate and Surgical Intervention on Adolescent Life Outcomes
Source: Ann Glob Health. 2022 Apr 13;88(1):25. doi: 10.5334/aogh.3679 (PMC9009361; doi:10.5334/aogh.3679)
Supplement: Appendix. — Additional regression tables and consent forms. [file agh-88-1-3679-s1.pdf]

Appendix

Table A1: Regional Fixed Effects Estimates

Panel A:	Speech Index	Physical Wellbeing	Social Integration	Psychological Wellbeing	Academic and Cognitive Index	Human Dignity Index
Cleft Severity	-0.310***<br>(0.0334)	-0.0835***<br>(0.0163)	-0.0382*<br>(0.0194)	-0.0206<br>(0.0253)	-0.0609**<br>(0.0241)	-0.0629***<br>(0.0175)
Cleft Surgeries	-0.163**<br>(0.0668)	0.0944**<br>(0.0407)	-0.0223<br>(0.0411)	-0.0728<br>(0.0540)	0.0206<br>(0.0443)	-0.0223<br>(0.0317)
First Surgery ≤ Five Years Old	0.305*<br>(0.160)					
N	954	1118	1118	1118	1118	1118

Panel B:	Speech Index	Physical Wellbeing	Social Integration	Psychological Wellbeing	Academic and Cognitive Index	Human Dignity Index
Cleft Severity	-0.306***<br>(0.0343)	-0.0834***<br>(0.0168)	-0.0355*<br>(0.0205)	-0.0226<br>(0.0260)	-0.0644**<br>(0.0236)	-0.0650***<br>(0.0176)
Cleft Surgeries	-0.143**<br>(0.0696)	0.0947**<br>(0.0406)	0.0000978<br>(0.0393)	-0.0889<br>(0.0558)	-0.00723<br>(0.0418)	-0.0400<br>(0.0371)
Operation Smile Surgeries	-0.115<br>(0.150)	-0.00166<br>(0.0629)	-0.108<br>(0.116)	0.0776<br>(0.110)	0.134<br>(0.0859)	0.0850<br>(0.0943)
First Surgery ≤ Five Years Old	0.315*<br>(0.169)					
N	954	1118	1118	1118	1118	1118

OLS with fixed effects at the regional level that include sibling controls for gender, age, and birth order, a dummy variable indicating whether a household has a CLP child, education and occupation of parents, housing quality index, and dummy variables indicating if a household is Christian or Muslim (default = Hindu). Standard errors clustered at the region level and are in parentheses. Dependent variables are all standardized indices in the manner of Kling et al.[13] $^{*}p < 0.10$, $^{**}p < 0.05$, $^{***}p < 0.01$.

Table A2: Mediation Tests for Social Inclusion

	Social Inclusion Index:					
Born w/Cleft Lip	-0.233**		-0.216**	0.105		0.0401
	(0.0938)		(0.0983)	(0.220)		(0.249)
Unoperated (Visible) Cleft Lip		-0.384	-0.164	-0.235		-0.0851
		(0.327)	(0.344)	(0.347)		(0.288)
Cleft Severity				-0.0660		0.0341
				(0.0410)		(0.0546)
Speech Quality					0.180***	0.251***
					(0.0459)	(0.0768)
	1109	1118	1109	1109	954	947

OLS with fixed effects at the household level. Standard errors clustered at the household level and are in parentheses. Regressions control for individual variables including gender, birth order, and age. Dependent variables are all standardized Kling et al.[13] indices. $*p < 0.10$, $**p < 0.05$, $***p < 0.01$.

Table A3: Effects of Surgeries by Type and Provider

	Speech Index	Physical Wellbeing	Social Integration	Psychological Wellbeing	Academic/ Cognitive	Human Flourishing
Required Surgeries	-0.341***	-0.076***	-0.0602*	-0.0469*	-0.0928***	-0.0873***
	(0.0367)	(0.0213)	(0.0351)	(0.0270)	(0.0284)	(0.0264)
Number of Cleft-Lip Surgeries	-0.134	0.0878	0.0749	-0.0634	-0.0241	-0.00845
	(0.133)	(0.0760)	(0.114)	(0.0937)	(0.0926)	(0.0843)
Number of Cleft Palate Surgeries	0.0748	-0.0250	0.158	-0.0612	0.0209	0.00400
	(0.0996)	(0.0744)	(0.101)	(0.0936)	(0.0733)	(0.0771)
Number of OS Cleft-Lip Surgeries	0.119	0.0815	-0.0741	0.429***	0.306**	0.219
	(0.163)	(0.110)	(0.171)	(0.158)	(0.125)	(0.138)
Number of OS Cleft-Palate Surgeries	-0.311*	-0.0246	-0.384**	-0.167	0.139	-0.126
	(0.166)	(0.116)	(0.170)	(0.160)	(0.152)	(0.143)
N	954	1118	1118	1118	1118	1118

OLS with fixed effects at the household level. Standard errors clustered at the household level and are in parentheses. Regressions control for individual variables including gender, birth order, and age. Dependent variables are all standardized Kling et al.[13] indices. *$p < 0.10$, **$p < 0.05$, ***$p < 0.01$.

INFORMED CONSENT FORM
UNIVERSITY OF SAN FRANCISCO
CONSENT TO BE A RESEARCH SUBJECT
-For adults 18 and older-

Purpose and Background

My name is ______________________ and I am a research assistant working on behalf of researchers at the University of San Francisco in the USA. I am asking you to participate in a project that examines the impact of receiving cleft lip and cleft palate surgery. Our study aims to measure the impact on a range of outcomes that may be affected by access to reparative cleft surgery and taking part in this survey will help us know the true value of receiving surgery as a young child.

Procedures
If I agree to allow my child to participate in this study, the following will happen:
1. I will complete a half hour survey conducted by the researchers and their assistants. I will be asked information about my age, gender, education, and information about each of my children.

Duration and Location of the Study
My family's participation in this study will involve one session that lasts up to 2 hours. The study will take place in West Bengal, India. My child may quit this study at any time by simply saying "Stop" or "I do not wish to participate."

Confidentiality

Any data my child provides in this study will be kept confidential unless disclosure is required by law. In any report we publish, we will not include information that will make it possible to identify you or any individual participant. Specifically, we will transfer survey information onto a password-protected computer and remove all identifying information. Although the researchers may ask for my child's name during the interview, all identifying information will be coded as numbers in a way that does not allow researchers to distinguish one participant from another.

Risks and Discomforts
There are no expected risks or comforts associated with taking part in this study.

Payment/Reimbursement
My family will be reimbursed 1,000 rupees for their full participation in this study. I will be paid in cash immediately after each member of my family (one parent/guardian, child with cleft, and their closest age sibling) has completed the survey. If any member of my family decides to withdraw from the study, I will not receive payment. If the researchers decide to terminate study participation, my family will still receive the full reimbursement.

Questions
I have talked to one of the research assistants about this study and have had my questions answered. If I have any question about this study, please contact any of the following researchers: Kira Evsanaa (khatansuudal@yahoo.com), Jeremiah Maller (rjmaller@gmail.com), or Sam Manning (sam.j.manning@gmail.com)

PARTICIPATION IN RESEARCH IS VOLUNTARY

I am free to decline to have my child be in this study, or to withdraw my child from it at any point. My decision as to whether or not to have my child participate in this study will have no influence on the surgery or medical services my child receives and my family will be paid 1,000 rupees even if the respondent chooses not to answer some questions. My signature below indicates that I agree to allow my child to participate in this study. In addition, the researcher has the right to withdraw my child from participation in the study at any time.

I HAVE READ THE ABOVE INFORMATION. ANY QUESTIONS I HAVE ASKED HAVE BEEN ANSWERED. I AGREE TO PARTICIPATE IN THIS RESEARCH PROJECT AND I WILL RECEIVE A COPY OF THIS CONSENT FORM.

____

Child's Name (print clearly)

____

Signature of Subject's Parent/Guardian *Date*

____

Signature of Person Obtaining Consent *Date*

REQUESTING ASSENT FOR AN OLDER CHILD
UNIVERSITY OF SAN FRANCISCO
-For respondents under the age of 18-

Dear Sir/Mam,

My name is ______________________ and I am a research assistant working on behalf of researchers at the University of San Francisco in the USA. I am asking you to participate in a project that examines the impact of receiving cleft lip and cleft palate surgery. Our study aims to measure the impact on a range of outcomes that may be affected by access to reparative cleft surgery and taking part in this survey will help us know the true value of receiving surgery as a young child.

I am asking you to complete a questionnaire that may take about 45 minutes. Your parents or legal guardians have already given a permission for you to participate in this study, but you do not have to participate if you choose not to. You may quit this study at any time by simply telling us that you do not want to continue. You can skip any questions or tasks that you do not want to complete. There are no known risks involved in this study. Conditional on you, your nearest age sibling, and one of your parents taking part in this study, you and your family will receive 1000Rs. total. If you or one of your family members decides to withdraw from the study, you will not receive 1000Rs. If the researchers decide to terminate the study, you and your family will receive 1000Rs.

To protect your confidentiality, your responses will not be shared with anyone unless required by law. The responses you give will be kept by the research team on a password-protected computer. Aside from the research team, nobody will know if you choose to participate in this project, nor will anyone know the answers you provide.

If you have any question about this study, please contact any of the following researchers: Kira Evsanaa (khatansuudal@yahoo.com), Jeremiah Maller (rjmaller@gmail.com), or Sam Manning (sam.j.manning@gmail.com)

Agreement

I agree to participate in this research project, and I have received a copy of this form.

____

Participant's Name (Please Print)

____

Participant's Signature *Date*

I have explained to the above-named individual the nature and purpose, benefits and possible risks associated with participation in this research. I have answered all questions that have been raised and I have provided the participant with a copy of this form.

____

Signature of Person Obtaining Consent *Date*

PARENTAL CONSENT FORM
UNIVERSITY OF SAN FRANCISCO
PARENTAL CONSENT FOR RESEARCH PARTICIPATION
-For parents of survey respondents under the age of 18-

Purpose and Background

My name is ______________________ and I am a research assistant working on behalf of researchers at the University of San Francisco in the USA. I am asking you to participate in a project that examines the impact of receiving cleft lip and cleft palate surgery. Our study aims to measure the impact on a range of outcomes that may be affected by access to reparative cleft surgery and taking part in this survey will help us know the true value of receiving surgery as a young child.

Procedures

If I agree to allow my child to participate in this study, the following will happen:
1. I will complete a half hour survey conducted by the researchers and their assistants. I will be asked information about my age, gender, education and information about each of my children.
2. The researchers will review my child's medical records to obtain information about the nature and extent of my child's cleft lip or palate.
3. The researchers will ask my child to complete a 45-minute survey to answer questions about age, gender, school, their social life, and their health.

Duration and Location of the Study

My family's participation in this study will involve one session that lasts up to 2 hours. The study will take place in West Bengal, India. My child or I may quit this study at any time by simply saying "Stop" or "I do not wish to participate."

Confidentiality

Any data my child provides in this study will be kept confidential unless disclosure is required by law. In any report we publish, we will not include information that will make it possible to identify you or any individual participant. Specifically, we will transfer survey information onto a password-protected computer and remove all identifying information. Although the researchers may ask for my child's name during the interview, all identifying information will be coded as numbers in a way that does not allow researchers to distinguish one participant from another.

Risks and Discomforts

There are no expected risks or comforts associated with taking part in this study.

Payment/Reimbursement

My family will be reimbursed 1,000Rs. for their participation in this study. I will be paid in cash immediately after each member of my family (one parent/guardian, child with cleft, and their closest age sibling) has all completed the survey. If my child or any member of my family decides to withdraw from the study before I have completed participating, I will not receive the reimbursement. If the researchers decide to terminate the study participation, my family will receive the full reimbursement.

Questions

I have talked to one of the research assistants about this study and have had my questions answered. If I have any question about this study, please contact any of the following researchers: Kira Evsanaa (khatansuudal@yahoo.com), Jeremiah Maller (rjmaller@gmail.com), or Sam Manning (sam.j.manning@gmail.com)

PARTICIPATION IN RESEARCH IS VOLUNTARY

I am free to decline to have my child or myself to be in this study, or to withdraw my child and myself from it at any point. My decision as to whether or not to have my child participate in this study will have no influence on the surgery or medical services my child receives and my family will be paid 1,000Rs. even if the respondent chooses not to answer some questions. My signature below indicates that I agree to allow my child to participate in this study. In addition, the researcher has the right to withdraw me and my child from participation in the study at any time.

I HAVE READ THE ABOVE INFORMATION. ANY QUESTIONS I HAVE ASKED HAVE BEEN ANSWERED. MY SIGNATURE BELOW INDICATES THAT I AGREE TO ALLOW MY CHILD TO PARTICIPATE IN THIS STUDY

____

Child's Name (print clearly)

____

Signature of Subject's Parent/Guardian *Date*

____

Signature of Person Obtaining Consent *Date*
